# Social Activity and All-Cause Mortality in Older Black Adults of the Minority Aging Research Study

**DOI:** 10.1093/geroni/igab046.1000

**Published:** 2021-12-17

**Authors:** Melissa Lamar, Bryan James, Ana Capuano, Robert Wilson, Lisa Barnes

**Affiliations:** 1 Rush Alzheimer's Disease Center, Rush University Medical Center, Rush Alzheimer's Disease Center, Illinois, United States; 2 Rush University, Chicago, Illinois, United States; 3 Rush, Chicago, Illinois, United States; 4 Rush University Medical Center, Chicago, Illinois, United States

## Abstract

Negative social stressors (e.g., perceived loneliness and/or social isolation) predict mortality in older adults; less is known about the role of positive social activities. What research does exist focuses on White adults, leaving gaps in knowledge regarding specific activities that may decrease mortality risk in Black adults. We investigated whether self-reported late-life social activity, generally and by type, predicted mortality risk in 768 Black adults (age~73yrs; non-demented at baseline) participating in MARS. Over ~6.5 follow-up years, 25% of participants died (n=192; age-at-death~83yrs). In fully-adjusted Cox models including demographic, health, and relevant psychosocial covariates, mortality risk decreased by 32% (HR=0.68,95%CI=0.49,0.93) in those with higher compared to lower social activity generally, and with higher volunteer-, church- and group-related activities specifically. Engaging in social activity, especially altruistic or faith-based activities, reduces mortality risk in older Blacks regardless of overall health or social stressors pointing toward community-based approaches to increase longevity in this population.

